# Electrophysiological Frequency Band Ratio Measures Conflate Periodic and Aperiodic Neural Activity

**DOI:** 10.1523/ENEURO.0192-20.2020

**Published:** 2020-12-17

**Authors:** Thomas Donoghue, Julio Dominguez, Bradley Voytek

**Affiliations:** 1 Department of Cognitive Science, University of California, San Diego; 2 Halıcıoğlu Data Science Institute, University of California, San Diego; 3 Neurosciences Graduate Program, University of California, San Diego; 4 Kavli Institute for Brain and Mind, University of California, San Diego, La Jolla, CA 92093

**Keywords:** aperiodic neural activity, frequency band ratios, neural oscillations, spectral analyses, θ/β ratio

## Abstract

Band ratio measures, computed as the ratio of power between two frequency bands, are a common analysis measure in neuroelectrophysiological recordings. Band ratio measures are typically interpreted as reflecting quantitative measures of periodic, or oscillatory, activity. This assumes that the measure reflects relative powers of distinct periodic components that are well captured by predefined frequency ranges. However, electrophysiological signals contain periodic components and a 1/f-like aperiodic component, the latter of which contributes power across all frequencies. Here, we investigate whether band ratio measures truly reflect oscillatory power differences, and/or to what extent ratios may instead reflect other periodic changes, such as in center frequency or bandwidth, and/or aperiodic activity. In simulation, we investigate how band ratio measures relate to changes in multiple spectral features, and show how multiple periodic and aperiodic features influence band ratio measures. We validate these findings in human electroencephalography (EEG) data, comparing band ratio measures to parameterizations of power spectral features and find that multiple disparate features influence ratio measures. For example, the commonly applied θ/β ratio is most reflective of differences in aperiodic activity, and not oscillatory θ or β power. Collectively, we show that periodic and aperiodic features can create the same observed changes in band ratio measures, and that this is inconsistent with their typical interpretations as measures of periodic power. We conclude that band ratio measures are a non-specific measure, conflating multiple possible underlying spectral changes, and recommend explicit parameterization of neural power spectra as a more specific approach.

## Significance Statement

Neural oscillations are a ubiquitous feature of investigation in electrophysiological recordings. Frequency band ratio measures are a common approach to investigate neural oscillations, applied across cognitive and clinical neuroscience, and in recording modalities such as in electroencephalography and local field potentials. In this work, we systematically investigate the methodological properties of band ratio measures. We show that band ratio measures are not specific to measuring oscillatory power, as they are intended and interpreted to do. Rather, they often reflect other features of the data, such as aperiodic, or 1/f-like, activity. These findings are significant for interpreting prior empirical and clinical research, guiding future work, and another motivation that aperiodic neural activity should be a key consideration when studying electrophysiological data.

## Introduction

Frequency band ratio measures, in which a ratio of power is calculated between prespecified frequency bands, are a common analysis measure in cognitive and clinical neuroscience. For example, a consistent line of research investigates the θ/β ratio as a potential biomarker for executive function, and in particular attentional processing (Lubar, 1991; Angelidis et al., 2016; Gordon et al., 2018; van Son et al., 2019). Other work has explored using ratio measures in learning and memory (Nokia et al., 2008; Kim et al., 2016; Trammell et al., 2017), age-related changes (Matoušek and Petersén, 1973; Gasser et al., 1988; Clarke et al., 2001), and automated sleep scoring (Costa-Miserachs et al., 2003; Krakovská and Mezeiová, 2011; Reed et al., 2017).

Band ratio measures are also common in clinical neuroscience, in studies seeking biomarkers for diagnosis, clinical monitoring, and potential intervention. Band ratio measures are commonly used in investigations of attention-deficit hyperactivity disorder (Lubar, 1991; Snyder and Hall, 2006; Loo and Makeig, 2012; Arns et al., 2013). Other investigations into the potential clinical utility of band ratio measures include anesthesia (Long et al., 1989), multiple sclerosis (Keune et al., 2017), cerebral ischemia (Sheorajpanday et al., 2009), and Parkinson’s disease (Geraedts et al., 2018). Band ratio measures have also been applied in studies of mild cognitive impairment, dementia, and Alzheimer’s (Penttilä et al., 1985; Bennys et al., 2001; Moretti et al., 2013; for recent review, see Cassani et al., 2018) and have also been applied in studies of autism (Wang et al., 2016) and psychotic disorders (Howells et al., 2018).

**Figure 1.**
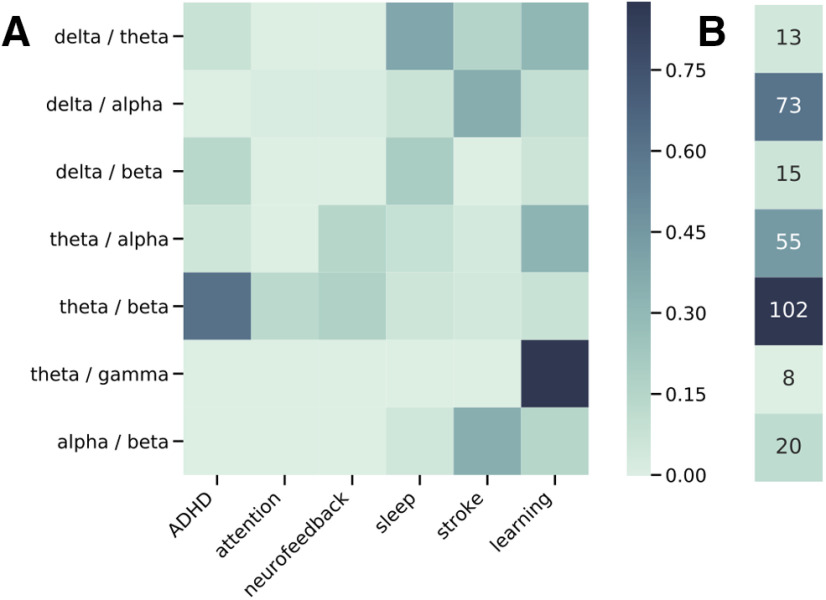
Literature analysis of band ratio related articles. ***A***, Associations between published journal articles referring to band ratio measures and cognitive and clinical associations. Each cell represents the proportion of articles referring to a specified band ratio measure that also mentions the corresponding association term. ***B***, Total counts of the number of articles mentioning each band ratio measure.

Collectively, band ratio measures are used across basic, clinical, and applied neuroscience. This is corroborated by an automated literature search that quantified the number of published articles that reference band ratio measures (Fig. 1), finding over 250 articles. Given the popularity of these measures, it is important to investigate their methodological properties and assumptions.

**Figure 2.**
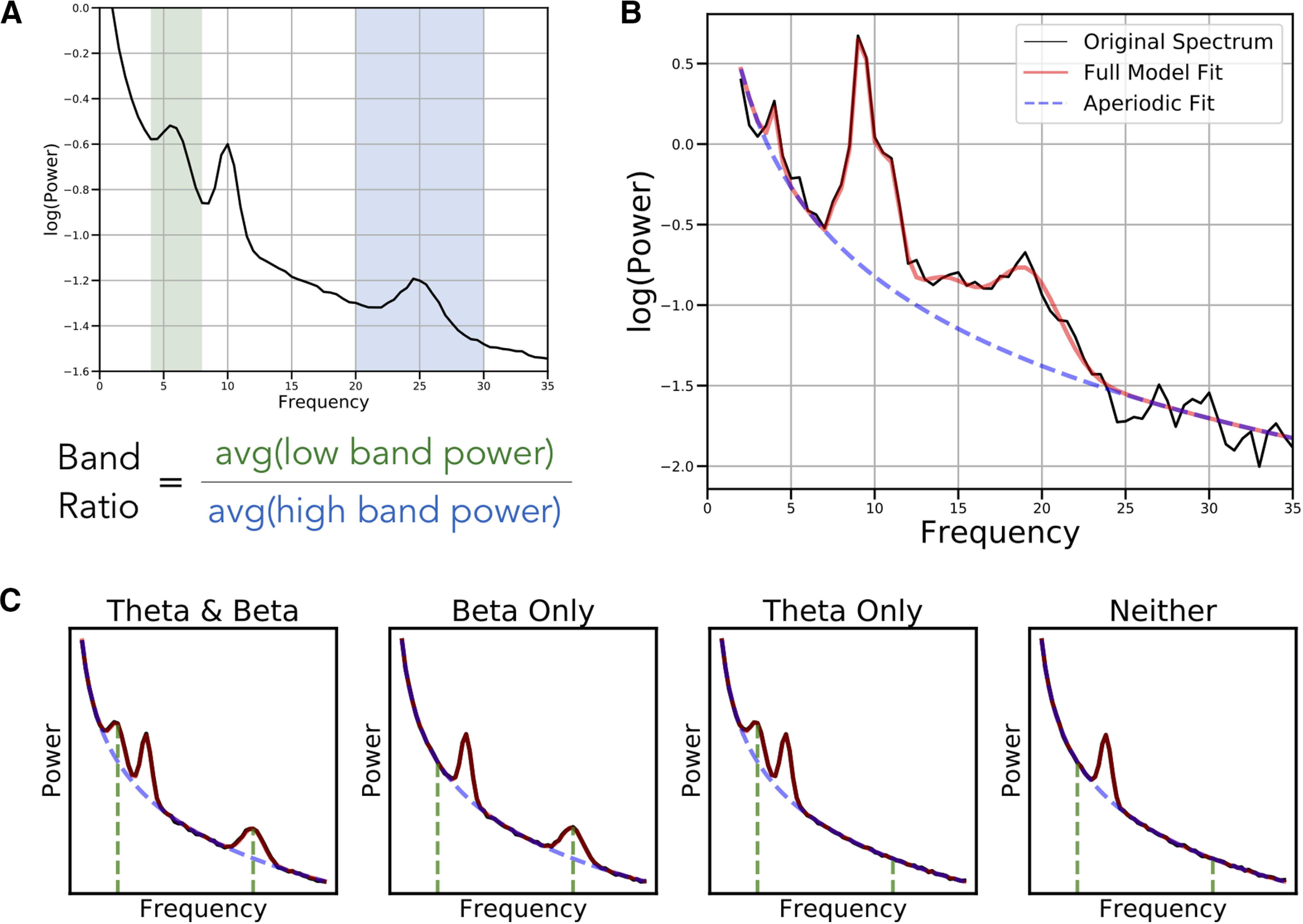
Overview of band ratio measures and spectral parameters. ***A***, An example power spectrum in which shaded regions reflect the θ band (4–8 Hz) and β band (20–30 Hz), respectively. Band ratio measures, such as the θ/β ratio, are calculated by dividing the average power between these two bands. ***B***, An example of a parameterized power spectrum, in which aperiodic activity is separated from measured periodic components. This is an example spectrum from EEG data, in which peaks in θ, α, and β power are present. ***C***, Examples of simulated power spectra with and without component oscillations of the θ/β ratio. Black lines indicate the simulated data, with red line reflecting the model fit, the dashed blue line indicating the aperiodic component of the model fit, and the green lines indicating the location of canonical θ and β oscillations. Band ratio measures, although intended to measure periodic activity, will reflect power at the predetermined frequencies regardless of whether there is evidence of periodic activity at those frequencies.

Studies using band ratio measures typically compute power in predefined frequency bands, and then calculate a ratio measure between them (see Fig. 2*A*). The result is then analyzed for potential correlations with features of interest. Such analyses typically interpret band ratio measures as reflecting periodic power, under the assumption that prespecified frequency bands specifically measure oscillatory activity.

**Figure 3.**
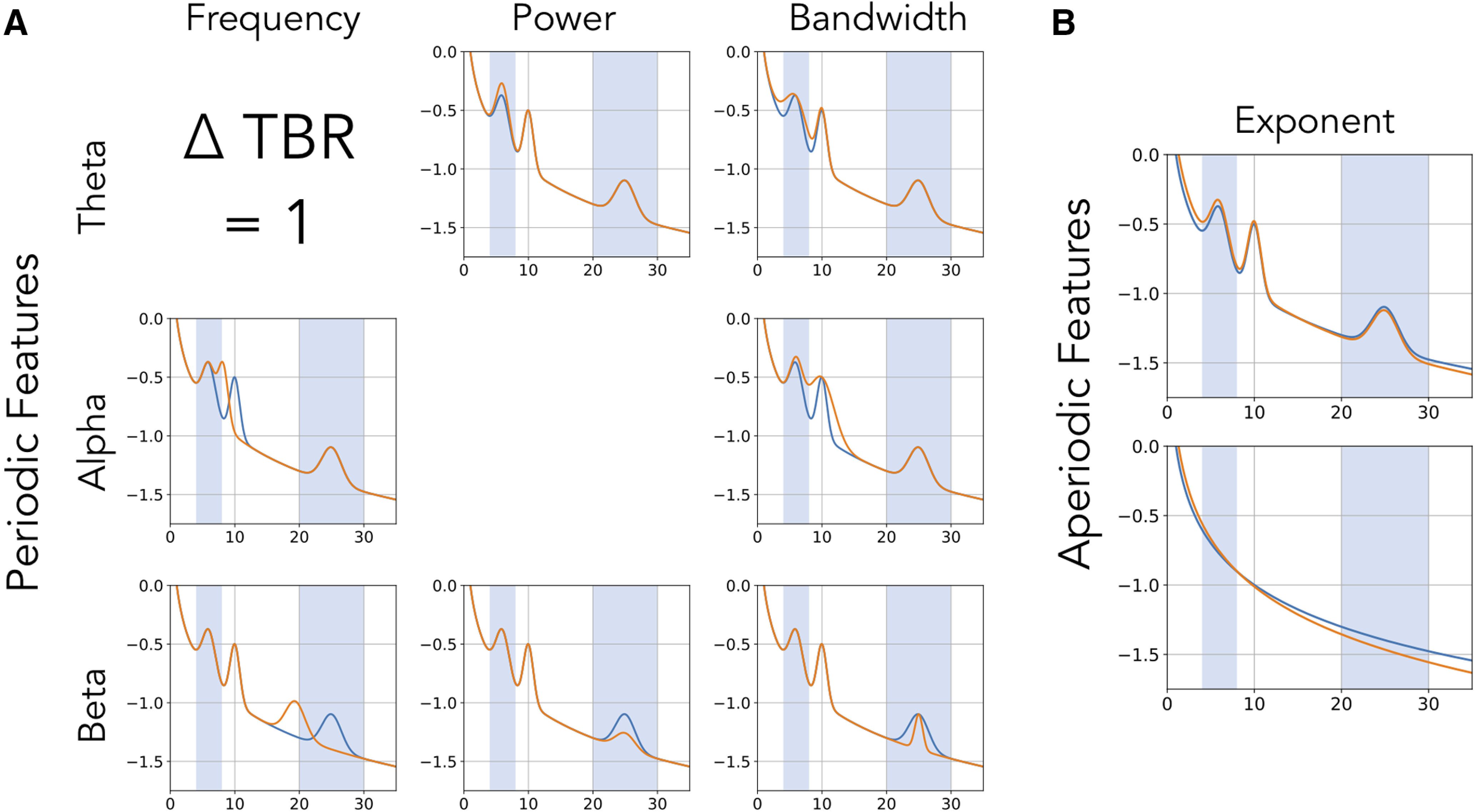
Equivalent band ratio differences from distinct changes. Simulations demonstrating the underdetermined nature of band ratio measures. In each case, the power spectrum plotted in orange has the same difference of measured θ/β ratio, indicated as Δ TBR, from the reference spectrum, in blue. This difference in ratio can arise from changes in multiple different features of the data, including a shift in: (***A***) periodic parameters such as the center frequency, power, or bandwidth of oscillations, and/or from a shift in; (***B***) aperiodic properties of the data, in this case the aperiodic exponent. Differences in aperiodic activity can induce differences in measured band ratios, even without any periodic components present (bottom panel).

However, a known problem with applying predefined frequency bands uniformly across all participants is that variation in center frequencies can lead to misestimations of band powers (Lansbergen et al., 2011). These potential confounds between different periodic features of the data challenge the notion that band ratio measures relate specifically to periodic power (see Fig. 3*A*).

A broader issue is the implicit assumption that predefined frequency bands reflect only periodic activity, and that measuring the average power of a frequency range specifically captures periodic power. This assumption is in general invalid, as electrophysiological activity includes not only periodic components but 1/f-like aperiodic activity (He, 2014; Donoghue et al., 2020). This 1/f-like activity, henceforth referred to as the “aperiodic component,” has power at all frequencies, meaning there will always be power in a given frequency range, but is not comprised solely of periodic activity (see Fig. 2*B*).

Therefore, power in a given frequency range reflects, at least in part, aperiodic activity and only partially, if at all, periodic activity. A marker that there is actual periodic power in a signal is that there should be a band specific peak over and above this aperiodic component (Buzsáki et al., 2013). To specifically measure this periodic component of the signal, one should measure the power of overlying peaks relative to the aperiodic component of the signal (Donoghue et al., 2020). Band ratio measures, as currently applied, do not address the confound of ubiquitous aperiodic activity in neural signals. Aperiodic neural activity is known to be variable both within (Podvalny et al., 2015) and between (Voytek et al., 2015) individuals, which raises the possibility that band ratio measures may capture and reflect differences in aperiodic activity within and between individuals (see Fig. 3*B*).

In summary, band ratio measures are a common measure that are interpreted as reflecting periodic power. However, variations in periodic parameters and/or aperiodic activity, with or without oscillations even being present, can influence band ratio measures (Fig. 2*C*). This suggests that band ratio measures are underdetermined, whereby a change in one or many different features of the data may drive analogous differences in band ratio measures (Fig. 3). If so, typical interpretations of band ratio measures are unsupported, and band ratio measures may be uninterpretable, as there are many possible underlying causes of measured differences.

## Materials and Methods

In this investigation, we examined whether the conception of band ratios as measures that specifically reflect periodic power is supported. This question is motivated by considering that periodic properties of electrophysiological data are highly variable, often violating the assumptions of predefined frequency bands, and also that they also coexist with variable and dynamic aperiodic activity (Donoghue et al., 2020). To investigate this, we examined the properties and validity of band ratio measures, including (1) how are band ratio measures influenced by different features of periodic activity, including center frequency, power and bandwidth; and (2) how are band ratio measures influenced by changes in aperiodic properties of the data, including the aperiodic exponent and offset. To do so, we used both simulated data and an electroencephalography (EEG) dataset, and calculated band ratio measures, and compared these measurements to other quantifications of the data to investigate which properties of the data band ratio measures are sensitive to.

### Code accessibility

Analyses were done using Python (version 3.7), including common libraries numpy, pandas, scipy, matplotlib, and seaborn for analysis and visualization. The MNE library was used for managing and processing EEG data (Gramfort et al., 2014). Custom code was used to calculate band ratio measures and perform analyses. All code for this project is openly available in the project repository (https://github.com/voytekresearch/BandRatios) and in Extended Data 1.

10.1523/ENEURO.0192-20.2020.ed1 Extended Data 1 Project code. Supplementary package of code used for simulations and analysis. Download Extended Data 1, ZIP file.

### Literature analysis

The literature analysis was done using the Literature Scanner (LISC) Python toolbox (Donoghue, 2019). Briefly, this toolbox allows for collecting and analyzing literature data by curating search terms of interest, gathering related articles from available databases, and analyzing the results. For this analysis, a list of band ratio terms (e.g., “theta / beta ratio”) and related association terms (e.g., “attention”), with relevant synonyms and exclusion words, was manually curated. Searches were performed to determine the number of articles in the PubMed database that reference these terms in their abstract, and the number of cooccurrences of band ratio terms with association terms. Association scores were calculated as the proportion of articles referencing a band ratio measure that also mention one of the included association terms.

### Spectral measures

Band ratio measures are usually calculated from absolute power values, averaged across canonical frequency bands. For all analyses, canonical frequency bands were defined as: θ (4–8 Hz), α (8–13 Hz), β (13–30 Hz). In this study, band ratios were calculated from power spectra by dividing mean power across the low band range by the mean power across the high band range. For all analyses, we calculated the θ/β ratio, θ/α ratio, and α/β ratio. Ratio measures are often log-transformed, as they typically display a non-normal, skewed distribution. Where log-transformations of ratio values were used in analyses or visualizations, it is noted.

As a comparison to band ratio measures, periodic (oscillatory) and aperiodic properties of power spectra were characterized using the fitting-oscillations-and-one-over-f (FOOOF) toolbox (Donoghue et al., 2020) for parameterizing neural power spectra. Briefly, this tool measures both the aperiodic component of neural power spectra, described by the exponent and offset, and periodic peaks, described by the center frequency, power, and bandwidth of identified peaks. Band ratio measures were compared with the outputs of these parameterizations, to evaluate which parameters of the data the band ratio measures are sensitive to and primarily reflect. We also computed “parameterized ratios” which were ratio measures computed between the power of identified peaks from the parameterization procedure, as a measure of the ratio of isolated periodic power between bands, after controlling for aperiodic activity.

### Simulations

Neural power spectra were simulated to match the statistics of electrophysiological neural data, by combining a 1/f-like aperiodic component with overlying peaks of periodic activity, with overlying noise (Donoghue et al., 2020). The aperiodic component describes the 1/f-like characteristic of neural power spectra and is entirely described by the aperiodic “exponent” and “offset.” The aperiodic exponent, meaning the χ in $\frac{1}{f^{\chi }}$, describes the steepness of the 1/f, and the offset, describes the vertical translation of the aperiodic component. Periodic components describe putative oscillations that display power above the aperiodic component. Periodic components are simulated as Gaussians, and are described by a center frequency in hertz (Hz); peak power; over and above the aperiodic component, in arbitrary units (au); and bandwidth which describes the width of the peak, also measured in Hz. The simulation, for a power spectrum *P,* is described as the following:

$$P=L+\sum^{}G_{n},$$

in which *L* is the aperiodic component, described as the following:

$$L=b-\text{log}(f^{x}),$$

where b is the offset and χ is the exponent. Note that in these formulations, power is in log10 spacing. In linear power, the exponent would be written as 1/$f^{x}$, hence the label of one-over f.

Periodic components are added, whereby each peak is described as a Gaussian, as the following:

$$G_{n}=a*\text{exp}(\frac{-{\left(f-c\right)}^{2}}{2*w^{2}}),$$

in which *c* is the peak center frequency, and *a* and *w* are the height and width of the Gaussian, equivalent to the power and bandwidth of the peak. For both the aperiodic and periodic components, f is the array of frequencies of the power spectrum.

Spectra were simulated for the frequency range of 1–35 Hz, with 0.5 Hz frequency resolution. Default aperiodic and periodic parameter values were chosen to capture physiologically realistic values. These default values, as well as the ranges that parameters were simulated across for each parameter, for each frequency band, are given in Tables 1, 2. A small amount of normally distributed noise (0.005 au) was added per frequency to all spectra.

**Table 1:** Simulated periodic parameters

		θ	α	β
	Default	6	10	21.5
CF	Range	4–8	8–13	13–30
	Increment	0.25	0.25	1
	Default	0.5	0.5	0.5
PW	Range	0–1.0	0–1.0	0–1.0
	Increment	0.1	0.1	0.1
	Default	0.1	0.1	0.1
BW	Range	0.2–0.4	0.2–0.4	0.2–0.4
	Increment	0.2	0.2	0.2

Each parameter is given a default value, used when this parameter is included but not varied, and a range and increment, which define the range of simulated values when this parameter is systematically varied. CF: center frequency, PW: power, BW: bandwidth.

**Table 2:** Simulated aperiodic parameters

	Default	Range	Increment
Offset	0	0–2.5	0.25
Exponent	1	0–3	0.2

Same description as Table 1, for aperiodic parameters.

To measure how spectral parameters relate to band ratio measures, spectra were simulated where a single parameter was varied across a range while the remaining parameters were kept at their default values. From these spectra the θ/β, θ/α and α/β ratio measures were calculated to track how individual parameters relate to ratio measures. Since center frequency, power, and bandwidth are specific to a peak, they were individually varied for both low-band and high-band peaks.

We then studied how band ratio measures are affected by interacting changes in spectral parameters. Simulated power spectra were created where two parameters from the set {center frequency, power, bandwidth, exponent} were simultaneously varied across their respective ranges. All combinations of paired parameter simulations were calculated, and then analyzed by calculating band ratio measures and examining how simulated properties influence measured values. The default parameter settings and ranges remained the same as the single parameter simulations (as in Tables 1, 2).

### EEG data analysis

To further examine how various spectral parameters affect band ratio measures, in real data, we used the openly available Multimodal Resource for Studying Information Processing in the Developing Brain (MIPDB) dataset of human EEG data released by the Child Mind Institute (Langer et al., 2017). The study population is a community sample of children and adults (*n *=* *126, age range = 6–44, age mean = 15.79, age SD = 8.03, number of males = 69). Data for each subject includes resting state and task EEG data, behavioral measures, and eye tracking data. EEG data were collected on a 128 channel Geodesic Hydrocel system, from which the outermost channels, around the chin and neck, were excluded, leaving a standard 111 channel setup. For the current investigation, we analyzed eyes-closed resting state data. Of the 126 participants in the dataset, nine did not include resting state data collection, as indicated by the dataset description, and were therefore excluded. In addition, a further six participants were excluded from this analysis because of missing the resting state recording file (one subject) or not having enough resting data events to analyze (five participants) leaving 111 participants included in the final analysis.

In the resting state protocol, participants were instructed to fixate on a central cross, and open or close their eyes when they heard a beep, alternating between 20-s blocks of eyes open and 40-s blocks of eyes closed. The dataset includes a preprocessed and artifact corrected copy of the data, which was used here, with full details of the preprocessing described in Langer et al. (2017). Briefly, bad electrodes were identified and interpolated, eye artifacts were regressed out of the EEG from EOG electrodes, and a PCA approach was used to remove sparse noise from the data. We further identified flat channels (channels with no data) and interpolated them (average number of interpolated channels: 4.81 ± 0.15 SEM), and re-referenced data to a common average reference.

For the current analyses, we used the eyes closed resting state data, and extracted the time period of 5–35 s within the 40-s eyes closed resting segments, excluding the 5 s after and before eye opening. For the majority of the analyses, we used the first block for each participant. We also computed an analysis across blocks, in which power spectra and derived measures were computed separately for each of the five resting state blocks. Power spectra were calculated for each channel using Welch’s method, using 2-s windows with 25% overlap.

We then parameterized the calculated power spectra to return estimates of periodic and aperiodic parameters. The model parameterization we used is agnostic to frequency bands, fitting peaks wherever they are found in the frequency spectrum regardless of canonical band definitions (Donoghue et al., 2020). We determined that activity was contained in a band if the peak of an oscillation was contained in the aforementioned band definitions. Settings for parameterizing power spectra are as follows: the width for a detected peak was bound between 1 and 8 Hz, with a maximum number of detectable peaks set at 8, a minimum threshold for detecting a peak set at 0.1 au, the threshold for detecting was set at the default value of 2 SDs above the noise floor, and spectra were fit in “fixed” aperiodic mode, without a knee. Parameterizations were evaluated for quality, including manual checks, and using goodness-of-fit metrics, including the *r*^2^ between spectrum models and original data, which had mean value of 0.9732, indicating good fits.

### Statistical analyses

For all band ratio measures, we calculated Spearman correlations between spectral parameters, including center frequency, power, and bandwidth of each oscillation band, as well as the aperiodic exponent, across all channels. We do not report correlations to aperiodic offset, as offset shifts by themselves do not affect ratio measures (see simulation results). When analyzing between blocks, difference measures were computed as the measured value of each block, minus the measured value of the prior block, providing an estimate of how measured values vary across time. Spearman correlations were computed between measured ratios and spectral parameters, specifically with the parameterized peak powers and aperiodic exponent. In addition, we calculated Spearman correlations between each ratio measure and participants’ ages, and between spectral parameters and age.

For all computed correlations, we applied bootstrapping approaches to compute confidence intervals (CIs) for each reported measure and, where appropriate, to test the difference between correlation magnitudes (Wilcox, 2016). CIs were computed by resampling, with replacement, and computing correlations for each resample, which creates a distribution from which CIs can be computed. For all bootstraps, 5000 resamples were used, and 95% CIs were computed. In addition, differences between correlations were also evaluated using bootstrapping. To do so, differences of correlations were computed on resamples, creating a distribution of bootstrapped differences of correlations, which can be used to test whether the measured difference is significantly different from zero. The distribution of difference measures was used to compute an empirical *p* value, testing a two-sided comparison of if the measured value is significantly different from zero.

## Results

### Simulation results

**Figure 4.**
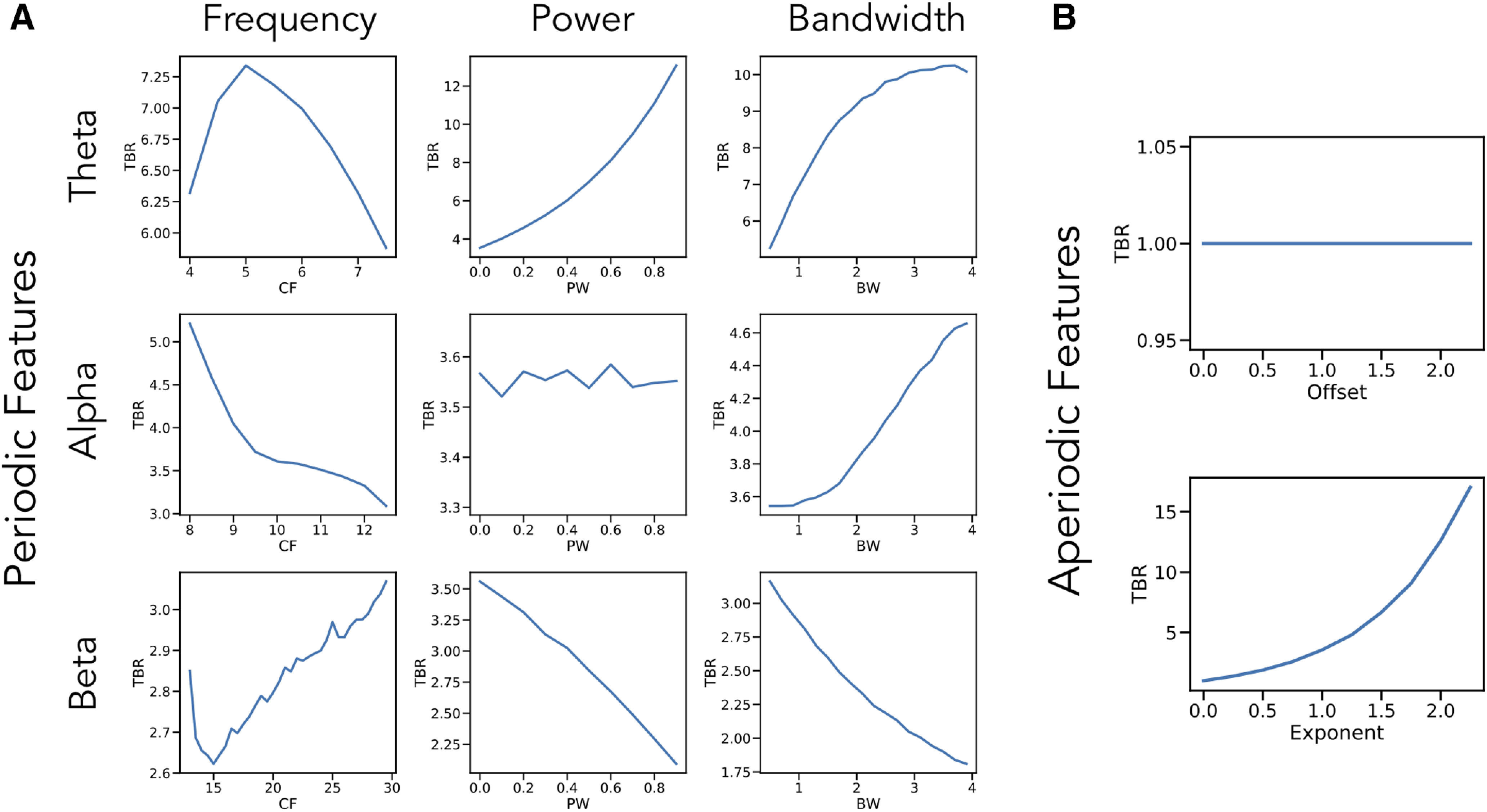
Single parameter simulations. Simulations of changes in measured θ/β ratio (TBR) as individual parameters are varied, including: (***A***) periodic parameters and (***B***) aperiodic parameters. Changes in θ center frequency show an increase in θ/β ratio as the heightened activity is better captured in the canonical band, then decreases as activity leaves the band. Increasing θ power and bandwidth both increase θ/β ratio while increasing β power and bandwidth decreases θ/β ratio. The center frequency and bandwidth of α peaks also influences measured θ/β ratio, although α is not supposed to be included in the measure. β parameters essentially have the inverse effect of changes in θ parameters. Changes in aperiodic exponent also substantially impact measured θ/β ratio, although offset has no effect. Note that the layout of this figure corresponds to Figure 3, in which examples of how each parameter influences measured θ/β ratio can be seen. CF: center frequency, PW: power, BW: bandwidth.

We started by investigating, in simulation, the extent to which band ratios capture periodic power as typically interpreted and/or to what extent they are potentially related to other periodic or aperiodic spectral parameters. Measured θ/β ratios across simulations in which one spectral parameter was changed at a time are reported in Figure 4. As expected, when examining periodic changes (Fig. 4*A*), the θ/β ratio is strongly driven by power of θ and β oscillations. However, ratio measures can also be influenced by the center frequency and bandwidth of the θ and β peaks. We also replicate previous work showing that the center frequency of the α peak can impact measures of θ/β ratio, (Lansbergen et al., 2011) and extend this to include α bandwidth. For aperiodic changes (Fig. 4*B*), we see that the aperiodic exponent has a significant effect on measured ratio values, but that the offset has no effect.

Collectively, we see that a wide range of different parameter changes can affect measured ratios. In this case, 8 of the 10 parameters alter θ/β band ratio, with the only exceptions being the aperiodic offset, which changes power equally between ratio bands, and power in the non-included band, in this case α (for the θ/β ratio). Of note, however, is that the scale of these effects can be quite different, with the power of the included bands and the aperiodic exponent having the biggest impacts. Simulations for other band ratio measures are consistent with those for the θ/β ratio, and are available in the project repository.

**Figure 5.**
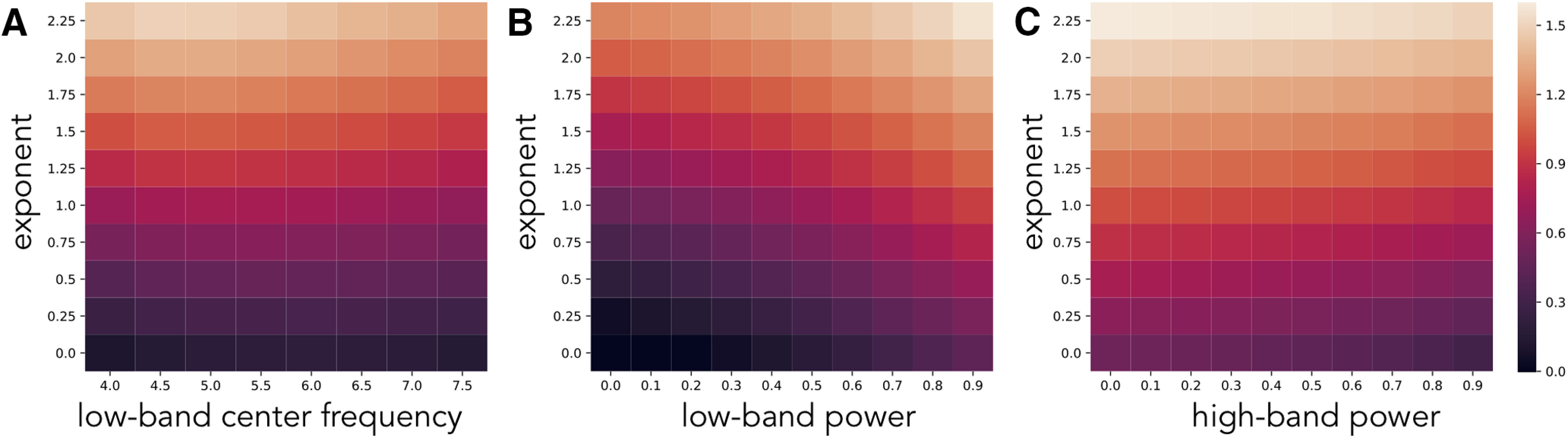
Interacting parameter simulations. Measured θ/β ratio values in simulations as two spectral parameters are varied together. Ratio measures are plotted in log10 space because of their skewed distributions. Combinations plotted are aperiodic exponent with low band center frequency (***A***), as well as with low band power (***B***), and high band power (***C***). All combinations of varying parameters influence measured band ratio values.

We further explored simulations of pairwise combinations of parameter changes, to investigate how ratio measures are affected by concomitant changes in multiple parameters (Fig. 5). These simulations include, for example, measured θ/β band ratios as the aperiodic exponent and θ center frequency both vary, showing an interaction between them (Fig. 5*A*). We can see how changes in aperiodic exponent interact with power changes in the lower (Fig. 5*B*) and higher (Fig. 5*C*) bands. These simulations also demonstrate that both features have an impact on measured ratios and allow a comparison of scale, showing, for example, that although the influence of low band power and aperiodic exponent is of a similar magnitude, when compared with high band power, the effect of aperiodic exponent changes is relatively much larger. Collectively, through these simulations, we see that changes in different spectral parameters can interact and drive different patterns of differences in measured band ratios. Further simulations of interacting parameters across all other combinations are available in the project repository.

### EEG data results

We next analyzed EEG data recorded during resting state and compared band ratio measures to parameterized power spectral features. For all analyses, we report results across all channels. Re-running these analyses with channel groups, using frontal, central, and parietal subselections all showed qualitatively similar patterns, the results of which are available in the project repository.

**Figure 6.**
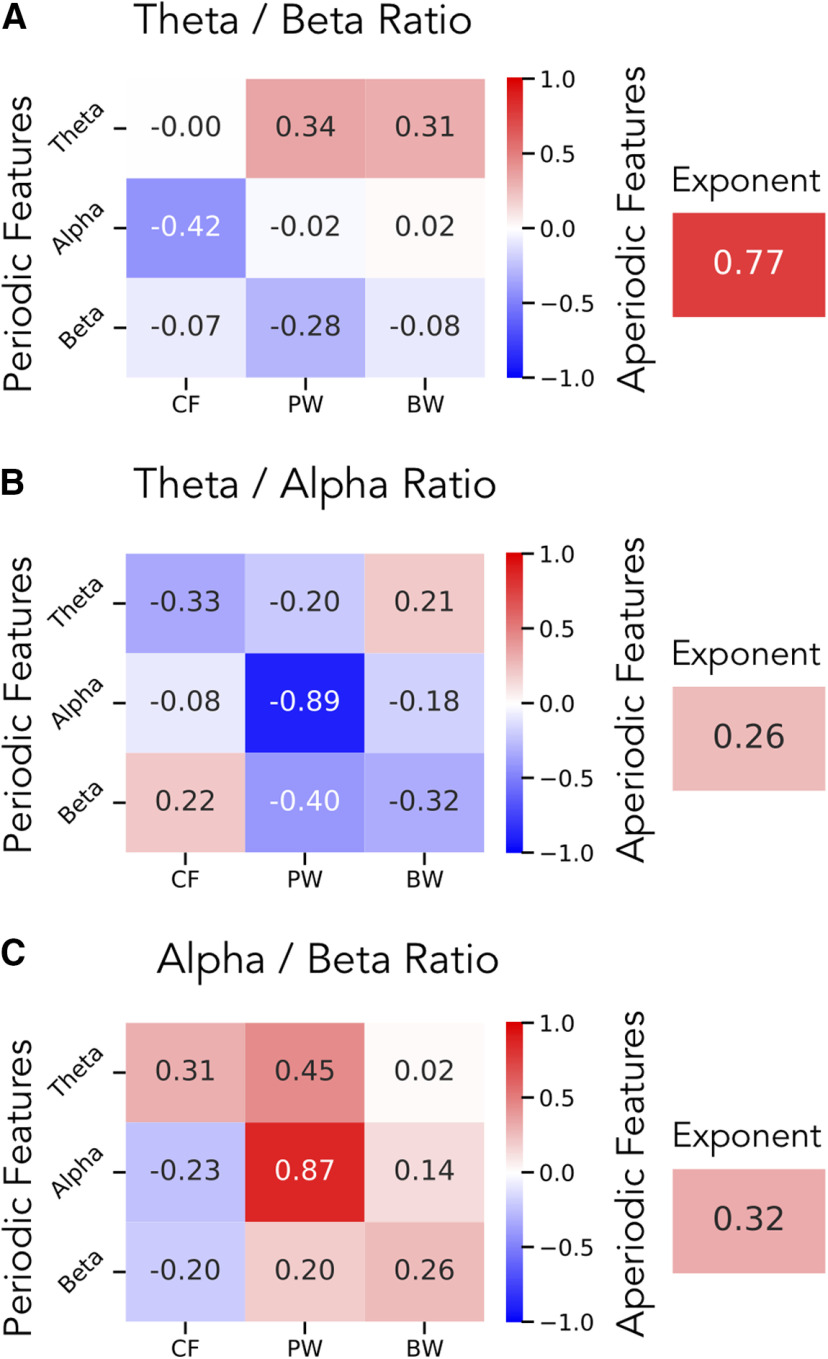
Correlations between spectral parameters and band ratio measures in EEG data. In a large EEG dataset, correlation results are reported for band ratios as compared with the periodic (left) and aperiodic (right) parameters for the (***A***) θ/β ratio, (***B***) θ/α ratio, and (***C***) α/β ratio. In ***A***, these results show that the θ/β ratio is most strongly correlated with the aperiodic exponent, and less related to power in the θ or β. In contrast, ***B***, ***C***, show that any ratio measure that includes an α band is most strongly correlated to α power, meaning any α ratio is mostly reflecting just α power. CF: center frequency, PW: power, BW: bandwidth.

For the θ/β ratio, within periodic spectral parameters, we find, as expected, that the strongest relationship is between θ/β ratio and θ power (*r *=* *0.34, CI_95_: [0.15, 0.52], *p *<* *0.001) with a similar magnitude correlation with β power (*r* = −0.28, CI_95_: [−0.46, −0.09], *p < *0.01). When ignoring direction (taking the absolute value of the correlations), the magnitude of the correlations between θ/β ratio and θ and β power is not significantly different (Δ*r *=* *0.06, CI_95_: [−0.25, 0.36], *p *=* *0.69). When considering aperiodic parameters, we find a much stronger relationship between θ/β ratio and aperiodic exponent (*r *=* *0.77, CI_95_: [0.66, 0.84] *p *<* *10^−20^). This correlation is of a significantly higher magnitude (ignoring direction) than the correlation to θ power (Δ*r *=* *0.42, CI_95_: [0.22, 0.62], *p *<* *10^−35^) or β power (Δ*r *=* *0.48, CI_95_: [0.26, 0.70], *p *<* *10^−35^). The full set of spectral parameter correlations is available in Figure 6*A*.

In contrast, for the θ/α ratio, the highest correlation across both periodic and aperiodic spectral parameters was for α power (*r* = −0.89, CI_95_: [−0.93, −0.84], *p *<* *10^−35^), with a much lower correlation to aperiodic exponent (*r *=* *0.26, CI_95_: [0.09, 0.42], *p *<* *0.01), with a significant difference of correlation magnitude between the two (Δ*r *=* *0.63, CI_95_: [0.45, 0.82], *p *<* *10^−35^). This pattern of correlations was also similar for the α/β ratio, with a strong correlation with α power (*r *=* *0.87, CI_95_: [0.79, 0.92], *p *<* *10^−30^), and a much weaker one with aperiodic exponent (*r *=* *0.32, CI_95_: [0.14, 0.49], *p *<* *0.001), again reflecting a significant difference in correlation magnitude (Δ*r *=* *0.54, CI_95_: [0.35, 0.73], *p *<* *10^−35^). Spectral parameter correlations for the θ/α ratio and α/β ratio are available in Figure 6*B*,*C*, respectively.

**Figure 7.**
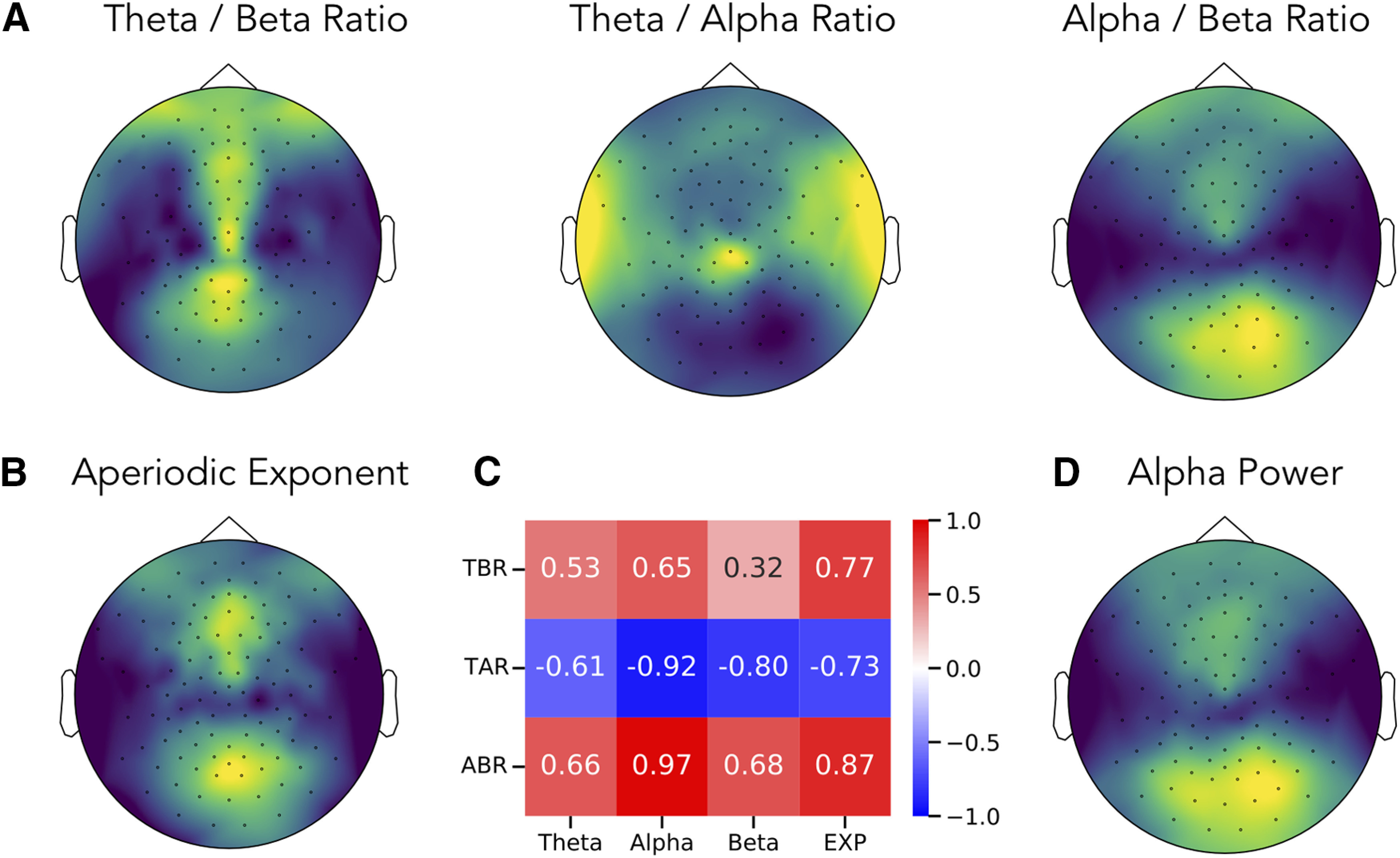
Topographies of band ratio measures and spectral parameters. Topographical maps of the (***A***) ratios measures, including the θ/β ratio, θ/α ratio and α/β ratio. For comparison, the topographies of the aperiodic exponent (***B***) and of α power (***D***) are also presented. Each topography is scaled to relative range of the data, with higher values plotted in lighter colors (yellow). ***C***, The spatial correlation between topographies of each ratio measure to spectral parameters including power of θ, α and β, and the aperiodic exponent (EXP). TBR: theta / beta ratio, TAR: theta / alpha ratio, ABR: alpha / beta ratio.

We also calculated average ratio measures and spectral parameters for each channel, across the group. Topographies of these measures are plotted in Figure 7. Here, we can see, for example, that the spatial topography of the θ/β ratio is most similar to that of the aperiodic exponent, with a strong spatial correlation (*r *=* *0.77, CI_95_: [0.66, 0.84], *p *<* *10^−20^) between them. Notably, the magnitude of the correlation of θ/β ratio to θ power (*r* = 0.53, CI_95_: [0.38, 0.64], *p* < 0.001) and β power (*r* = 0.32, CI_95_: [0.15, 0.48], *p* < 0.001) are both significantly less than the correlation of θ/β ratio to aperiodic exponent (θ power vs exponent: Δ*r* = −0.24, CI_95_: [−0.39, −0.11], *p* < 0.01; β power vs exponent: Δ*r* = −0.44, CI_95_: [−0.56, −0.33], *p* < 10^35^).

The topography of α/β ratio is nearly identical to the topography of α power (*r *=* *0.97, CI_95_: [0.95, 0.98], *p *<* *10^−70^). Similarly, there is a strong inverse relation between the θ/α ratio and α power (*r* = −0.92, CI_95_: [−0.95, −0.87], *p *<* *10^−45^). In these cases, the correlation of the θ/α ratio topography to α power was significantly greater than to aperiodic exponent (Δ*r* = −0.19, CI_95_:[−0.28, −0.11], *p* < 10^−35^), and the correlation between the α/β ratio topography and α power was also significantly greater than to aperiodic exponent (Δ*r *=* *0.11, CI_95_: [0.07, 0.17], *p* < 10^−35^).

**Figure 8.**
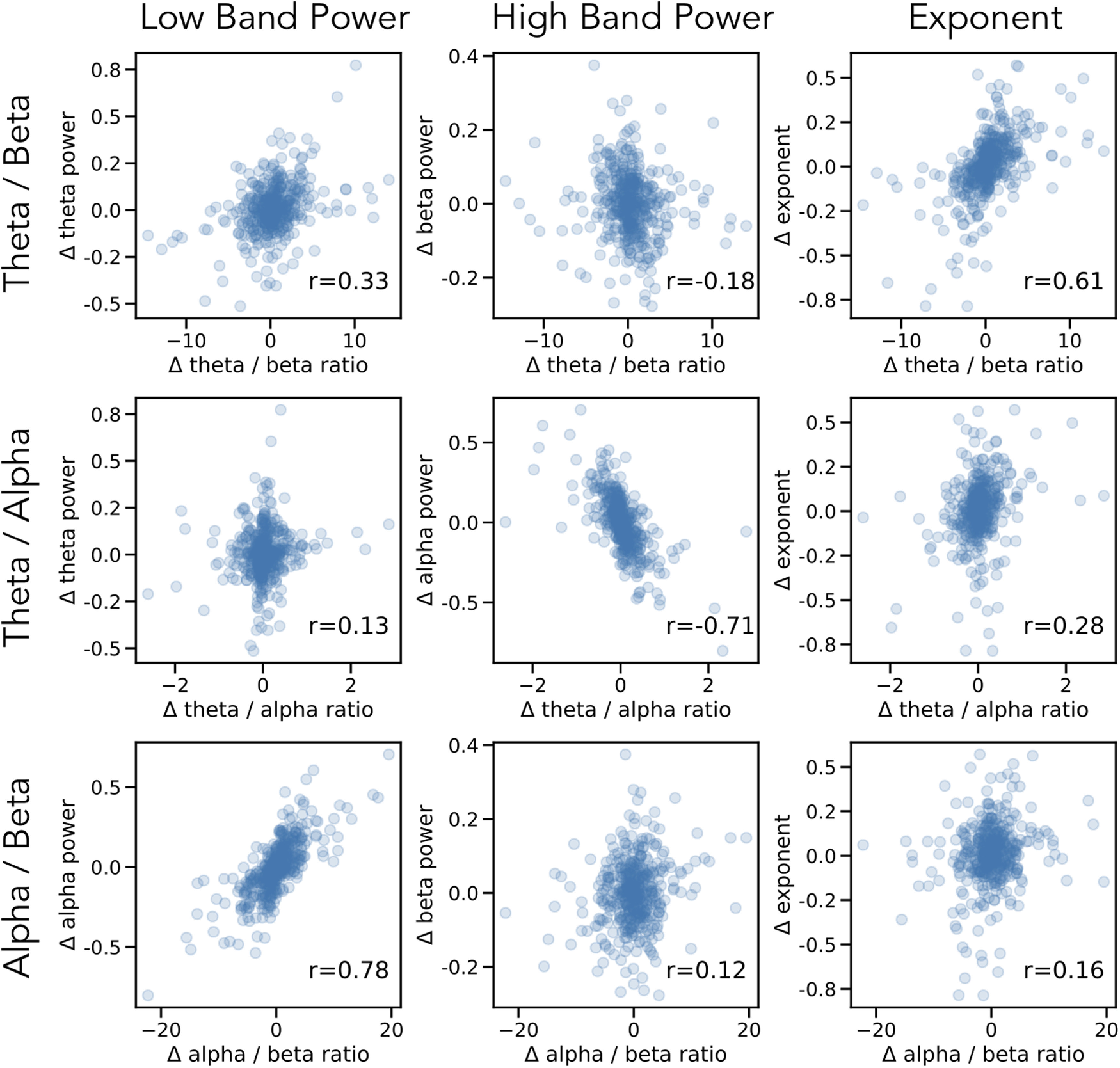
Changes in ratios and spectral parameters across blocks. Each row reflects a band ratio measure and each column reflects a spectral parameter. Each point is a difference measure across blocks, the value of the measure in a block, minus the value of that measure in the prior block, collected across all subjects. Printed in the inset is the spearman correlation between the measures. Consistent with prior analyses, changes across blocks in the θ/β ratio are most correlated with changes in aperiodic exponent, and changes in θ/α and α/β are most correlated with changes in α power.

We then examined how changes of each measure, across blocks, relate to each other. To do so, we correlated difference measures, calculated as the value of the current block minus the prior block, between ratio measures and spectral parameters (Fig. 8). We report the highest correlated parameter for each ratio, each of which had a significantly higher magnitude correlation than other parameters, as evaluated by bootstrap comparisons (see Materials and Methods). We find that variations across blocks of the θ/β ratio are highly correlated with variation of the aperiodic exponent (*r* = 0.61, CI_95_: [0.54, 0.67], *p* < 10^−44^). Variations of α power are mostly highly correlated with variations of the θ/α (*r* = −0.71, CI_95_: [−0.76, −0.64], *p* < 10^−66^) and α/β (*r* = 0.78, CI_95_: [0.73, 0.81], *p* < 10^−88^) ratios. These measures of variation, within subjects, across blocks, are consistent with the between-subjects analyses, showing dynamics of the θ/β ratio are related to dynamics of the aperiodic exponent, and that dynamics of ratios that include α are most related to α power.

We also calculated how each measure correlated with age. The θ/β ratio was found to be highly correlated with age (*r* = −0.67, CI_95_: [−0.76, −0.54], *p *<* *10^−15^), with the negative correlation indicating that older adults have lower θ/β ratios. The θ/α ratio also had a significant correlation with age (*r* = −0.37, CI_95_: [−0.51, −0.20], *p *=* *0.0001), but the α/β ratio was not significantly correlated with age (*r* = −0.12, CI_95_: [−0.30, 0.08], *p *=* *0.22). For spectral parameters, the aperiodic exponent was found to be highly correlated with age (*r *=* *0.68, CI_95_: [−0.77, −0.57], *p *<* *10^−15^), consistent with previous reports (Voytek et al., 2015; He et al., 2019). There was not a significant difference in the magnitude of the correlation of θ/β ratio and age and that of the aperiodic exponent and age (Δ*r *=* *0.01, CI_95_: [−0.01, 0.0], *p *=* *0.18).

We also calculated correlations between parameterized ratios (ratios computed on isolated periodic power) and age. We found that the parameterized θ/β ratio (*r* = −0.12, CI_95_: [−0.29, 0.05], *p *=* *0.21), parameterized θ/α ratio (*r* = −0.13, CI_95_: [−0.31, 0.05], *p *=* *0.18), and parameterized α/β ratio (*r* = −0.08, CI_95_: [−0.28, 0.11], *p *=* *0.38) were all non-significantly correlated with age. This is consistent with correlations between band ratio measures and age being driven by the influence of aperiodic activity, since no relation is found with isolated periodic power.

## Discussion

### Methodological discussion points

Through investigations of both simulated and real data, we find that frequency band ratio measures, although typically applied and interpreted as reflecting the relative periodic power of distinct frequency bands, can actually reflect a large number of distinct changes in the underlying data. These band ratio measures therefore capture multiple different changes in periodic and aperiodic properties. Part of this stems from the use of predefined frequency bands of interest, as has been previously reported (Lansbergen et al., 2011; Saad et al., 2018). Here, we replicate and extend this finding, showing how center frequency, and also oscillatory bandwidth, can influence band ratio measures in ways that can be misinterpreted as reflecting power differences. In addition, we show how frequency band ratio measures may commonly capture, at least partially, aperiodic components of electrophysiological data.

Specifically, we used a parameterization model conceiving of the power spectrum as the combination of an aperiodic, 1/f-like spectrum, characterized by an offset and exponent, with overlying periodic “peaks,” each characterized by a center frequency, power (over and above the aperiodic component), and bandwidth measure. With this approach, we show many of these parameters can affect band ratio measures in simulation. When applied to real data, we find that different parameters do affect ratio measures, with different patterns for different ratio measures. For example, θ/β ratio measures mostly reflect aperiodic exponent, whereas θ/α and α/β ratios mostly reflect α power. In no ratio measures did we find evidence that the measure primarily reflects power within both specified bands.

Given the underdetermined nature of band ratio measures in the face of multiple features of the data that may be changing, we conclude that band ratio measures are not an appropriate measure for characterizing electrophysiological data, at least not in isolation. This is because they are uninterpretable in terms of knowing which component(s) of the data they actually reflect. We therefore recommend complementary or alternate approaches, such as parameterizing neural power spectra (Donoghue et al., 2020). Such approaches allow for specifically measuring periodic and aperiodic components and therefore a more precise quantification and identification of which features of the data vary within and between individuals.

A prior recommendation, that attempts to address center frequency differences (Lansbergen et al., 2011), is that band ratio measures should use individualized frequency bands (Saad et al., 2018). It should be noted that the recommended approach, originally proposed by Klimesch (1999), is to use individualized bands based on an α band anchor point, whereby θ and β can be defined as below and above the observed α peak. Although this addresses some issues with varying α center frequency, it does not specifically establish whether there is a defined θ or β peak, over and above aperiodic power, nor does it identify specific center frequencies should such periodic activity be present. Because this approach also does not separate aperiodic from periodic power, individualized peak detection, especially when anchored to α peaks, is insufficient to address the problems highlighted here.

It has previously been reported that ratio measures are stable and have high test-retest reliability within individuals (Ohlund, 2000; Monastra et al., 2001; Angelidis et al., 2016). This is not necessarily in conflict with the finding here that band ratio measures may reflect many distinct features of the data; stable test-retest reliability merely suggests that whichever feature(s) are captured by band ratios within a given subject are themselves stable. However, that band ratios across individuals, and in particular across different populations, may reflect different properties of the data may well help explain why there has been difficulty in reproducing several findings using band ratios. For example, recent failures to replicate band ratio measures include follow ups on previously reported relations with trait anxiety (van Son et al., 2018) or attentional control (van Son et al., 2019). In clinical work, there have been inconsistent findings relating the θ/β ratio to ADHD (Ogrim et al., 2012; Liechti et al., 2013). It is possible that when investigating varying populations, different features of the data may be driving different observed ratio measures, and this may relate to the significant variance of band ratio measures and their correlates found across studies.

### Interpretation-related discussion points

Band ratio measures are often conceptualized as capturing the proportion of a “slower” frequency band relative to some “faster” one and are often interpreted as a relative “slowing” of neural activity (Monastra et al., 2001; Poza et al., 2008) or as a shift of power from one band to another (Gasser et al., 1988). Other interpretations focus on interpreting and investigating ratio measures in terms of changes within the component bands, for example, interpreting a decrease in θ/β ratio as changes in the θ or β band (Clarke et al., 2013), which conceptualizes one or more distinct changes in periodic bands. All of these conceptualizations consider that band ratios reflect periodic power.

In this work, we challenge the notion that ratio measures can be assumed to reflect periodic changes. While they can, and sometimes do, reflect changes in periodic power, they also reflect other parameters, and are often highly influenced by aperiodic activity. This is consistent with observations that helped motivate the use of band ratio measures, for instance, of correlated changes across frequency bands (Lubar, 1991). These observed correlated changes across frequency bands can be explained parsimoniously as a change in aperiodic activity. Changes in aperiodic exponent influences power across all frequencies and therefore induces correlations between any two measured frequency regions. This notion is somewhat consistent with the interpretations of ratios reflecting “substitutions” of power between bands (Gasser et al., 1988), in the sense that one process explains the changes across different frequency regions, although the conception that this is a shift of periodic activity is inconsistent with our findings.

These findings cast doubt on prior reports that use band ratio measures and interpret them as primarily reflecting periodic power. Where such studies are reproducible, recontextualization of such findings should consider multiple possible interpretations, including, for example, that (1) there is a true change in the power ratio of activity between distinct frequency bands reflecting periodic activity; (2) there is a difference in periodic parameters other than power, such as in center frequency and/or bandwidth; (3) there are differences in aperiodic activity; or (4) some combination of the above. Based on data analyzed, the θ/β ratio is most likely to reflect aperiodic activity, whereas the θ/α and α/β ratios are most likely to primarily reflect α power. That said, ratio measures could vary across studies in what they reflect and/or reflect interactions between parameters. Re-evaluations of prior work and/or follow-up investigations should seek to re-evaluate such data to investigate which features, in each case, are driving the measured changes in band ratios, and update interpretations accordingly.

In this investigation, we replicated the consistently reported finding that band ratio measures vary systematically with age (Gasser et al., 1988; Bresnahan et al., 1999; Clarke et al., 2001; Monastra et al., 2001; Putman et al., 2010; Ogrim et al., 2012; Buyck and Wiersema, 2014; Angelidis et al., 2016). We also replicate that aperiodic activity varies systematically with age (Voytek et al., 2015; He et al., 2019). The EEG dataset analyzed here consists of young participants, and the pattern of findings is also consistent with recent work showing that changes in aperiodic activity across age better explain developmental patterns compared with prior reports of correlated changes across multiple distinct oscillation bands (He et al., 2019). Since band ratio measures are highly correlated with aperiodic activity (especially the θ/β ratio), the relation of band ratios to age could be explained as a consequence of band ratio measures reflecting aperiodic activity. This interpretation is supported by the finding that parameterized ratios, using the isolated periodic power, do not correlate with age. The noted relation of band ratios to age is therefore likely to be a confound of aperiodic activity.

Overall, the EEG data analyzed here suggests that ratio measures, and the θ/β ratio in particular, often largely reflects aperiodic activity. As well as the relationship of aperiodic activity and band ratio measures to age, this is also consistent with other reports that find that correlates of band ratio measures may relate to aperiodic activity. For example, when band ratios are used in sleep scoring, it is typically done with the δ/θ ratio, which we predict likely also captures aperiodic changes. This would be consistent with recent reports that aperiodic activity changes systematically with sleep (Lendner et al., 2020). Collectively, these shared correlates are consistent with the suggestion that band ratio measures likely often reflect aperiodic activity.

A key prediction, if ratio measures often reflect aperiodic properties, is that the reported findings will not be specific to the frequency ranges used to measure the ratios, as aperiodic effects should exist across all frequencies. Indeed, correlated change across frequency bands is one of the observations that led to the popularity of band ratio measures (Lubar, 1991). It has also been reported that distinct ratio measures across different frequency bands show similar patterns, for example, that both δ/β and θ/β ratios relate to cognitive correlates (Schutter and Van Honk, 2005; Tortella-Feliu et al., 2014), both θ/α and θ/β have been reported to relate to ADHD (Barry et al., 2003), and multiple different ratios show similar patterns in investigations of Alzheimer’s disease (Poza et al., 2008). In cases such as these, in which different band ratio measures show approximately similar trends across a wide array of band pairs, a plausible interpretation is that these findings do not reflect correlated changes across multiple distinct frequency bands, but rather that they are all capturing frequency-agnostic aperiodic shifts.

Band ratio measures are also used as target for manipulation in neurofeedback paradigms. In such designs, findings are also consistent with the possibility that targeting ratios at least partially manipulates aperiodic properties, rather than targeting oscillation bands specifically. For example, a recent report showed that targeting β in a feedback design also induces power changes in the α band (Jurewicz et al., 2018), which challenges the possibility of targeting different bands independently. Where investigations probe the specificity of neurofeedback protocols, non-specific effects have been reported, such as an effect on β from a θ/α protocol (Egner et al., 2004), and changes in α when using a θ/β protocol (Yang et al., 2015; Bazanova et al., 2018), all of which is consistent with ratios reflecting aperiodic activity.

If a considerable proportion of the variance of band ratios measures is because of aperiodic properties, and not well described or interpreted as band specific changes, then it becomes an open question to ask what the physiological interpretation should be, and therefore how these findings should be interpreted. One hypothesis is that the aperiodic properties of neural time series may relate the relative balance of excitatory and inhibitory activity (Gao et al., 2017). Although further work is required to explore this hypothesis and how it relates to measurements done with band ratios, this does suggest a potential link between what has been measured in band ratios, as a correlate of various cognitive markers and disease states, and potential interpretations related to excitation and inhibition. A more general review of aperiodic properties in neural data, sometimes referred to “scale-free” activity, is available in He (2014).

In the case of ADHD, the θ/β ratio has been a focus of much research (for review, see Snyder and Hall, 2006; Arns et al., 2013), including being investigated as a potential diagnostic marker (Snyder et al., 2015). Findings have been inconsistent, with a reported lack of reliability across studies (Arns et al., 2013), and a practice advisory against using the θ/β ratio as a diagnostic marker for ADHD (Gloss et al., 2016). These inconsistent findings could potentially be explained by our findings, with the prediction that the θ/β measure is non-specific and inconsistent in how it is capturing different features of the data across subjects and studies, and that it is overall likely to be highly influenced by aperiodic activity. Indeed, it has recently been reported in a population of ADHD subjects that aperiodic properties are correlated with θ/β ratio measures, and that aperiodic measures better relate to disease state and medication status than any ratio measures (Robertson et al., 2019).

We therefore recommend that particular attention should be paid to ratio measures applied in clinical applications, in which the pursuit of biomarkers based on non-specific and unreliable measures could hinder, rather than ameliorate, clinical practice. For other clinical disorders that have been investigated with band ratio measures, such as Alzheimer’s disease (Cassani et al., 2018), or psychotic disorders (Howells et al., 2018), investigations should follow-up on which underlying features best explain changes in ratio measures, and update interpretations and future work on biomarkers accordingly.

A notable exception, as we found in analyzed EEG data, to ratio measures reflecting aperiodic shifts is in cases in which ratio measures include the α band. When the α band is included in the ratio, band ratio measures tend to primarily reflect α power. This is likely because of the prominence of the α band, where α is typically present across participants, has very high power, and is dynamic. Thus, it is logical that ratio measures that include the α band largely reflect α dynamics, as we observed here. This effect may also be exaggerated in our analysis, as we are analyzing eyes closed data, in which α power is most prominent, although the pattern of results is consistent when re-computed on eyes open data. Investigations in which ratio measures such as δ/α or θ/α are used should investigate to what extent the dominant effect they are capturing is α dynamics. Overall, we recommend that reports from studies using band ratios including α should consider whether the findings are likely to be largely explained by α dynamics.

## Conclusion

Frequency band ratio measures are a common analysis approach applied to neural field data, including EEG, MEG, ECoG, and LFP. Band ratio approaches have been applied across many domains, including in basic research investigating executive functions, learning and memory, and sleep; in clinical investigations including investigating ADHD and dementia; and in applied work leveraging them for neurofeedback applications. Although typically interpreted as a normalized measure reflecting the relative power of distinct periodic components, here we show that band ratio measures can reflect not only multiple features of periodic neural activity, including the center frequency, power, and bandwidth of periodic components, but can also be driven by variations in aperiodic activity. This is demonstrated both in simulation and in the analysis of a large EEG dataset, in which we show how multiple spectral features relate to measured band ratios, making them an imprecise metric. For example, the most dominant contributor to the θ/β ratio is the aperiodic exponent, whereas the θ/α and α/β ratios predominantly reflect α power. Overall, band ratio measures are found to be underdetermined, and so across participants, recording modalities, species, and contexts may reflect different components of the signal. This makes comparisons with band ratio measures difficult, if not impossible, and questions their typical interpretations as reflecting periodic activity. As an alternative, we recommend that parameterization of neural power spectra is able to better capture which components of neural signals vary and relate to features of interest, without conflating changes in periodic and aperiodic activity, as band ratio measures do.

## Synthesis

Reviewing Editor: Gustavo Deco, Universitat Pompeu Fabra Decisions are customarily a result of the Reviewing Editor and the peer reviewers coming together and discussing their recommendations until a consensus is reached. When revisions are invited, a fact-based synthesis statement explaining their decision and outlining what is needed to prepare a revision will be listed below. The following reviewer(s) agreed to reveal their identity: Luca Iemi.

Reviewer 1: In the present manuscript, the authors report that ratios of power estimates as derived from spectral EEG analyses do not necessarily reflect the ratio of two oscillatory processes. Instead they argue that several confounding factors -most prominently a change in the aperiodic, non-oscillatory component of the PSD (which exhibits a 1/f-like behavior)- can give rise to the previously observed changes in band ratios.

The manuscript is very timely and contributes to an emerging literature, which highlights issues with traditionally used spectral analyses. The manuscript is very well written and will constitute a valuable resource. A few minor aspects should be addressed:

1. A direct statistical comparison of different correlations should be conducted, given that the reported correlations from the empirical EEG data are dependent and not independent. Hence, the authors should use e.g. Steigers Test or a bootstrapping procedure (see also https://garstats.wordpress.com/2017/03/01/comp2dcorr/) to establish that correlations are significantly different. In its present form, it is statistically invalid to argue that correlation1 is stronger than correlation2 just based on the absolute value of the r statistic. A related issue is described e.g. in Nieuwenhuis 2011 Nature Neurosci.

2. The use of band ratios seems somewhat overstated; most groups that are concerned with network communication and neuronal oscillations do not utilize band ratios. In their literature research, the authors could also embed the number of citations (impact on the scientific landscape) in addition to simply stating that 250 papers utilized the metric.

3. The manuscript exhibits substantial overlap (also in terms of Figures, e.g. 2B) with other preprinted (e.g. Haller, Donoghue et al) manuscripts. While not explicitly spelled out in the other papers, these already imply that band ratios are strongly correlated with a change in the PSD slope. Furthermore, it is important to highlight that this paper (along with several others that are preprinted by the Voytek group) all rely on the FOOOF algorithm, which has not yet been published as a peer-reviewed paper.

4. On a more general note, it would be interesting how the fitting algorithm performs when one utilizes more 'optimal' spectral estimates than estimates as derived from Welchs method. In general, it should be pointed out that estimating band-ratios or the PSD slope really depends on a clean estimation of the underlying spectral distribution. For instance, Prerau and Brown 2017 argued for the use of multi-tapers to improve spectral estimation.

5. Regarding Fig 3, why is the reported Delta TBR = 1, isn't theta >> beta power, hence the ratio should be >> 1?

6. In Fig 4, what is the explanation for the impact of alpha on the reported metrics? It would be easier to assess why alpha power changes the theta/beta ratio if the authors could show the respective spectra. In addition, since the authors already suggest a certain directionality, they could try to incorporate a mediation analysis to demonstrate how changes in alpha power impact the PSD slope and thereby the theta/beta ratio - is it possible to establish (statistically) a directionality?

Taken together, the authors correctly alarm the field that band ratios are a suboptimal analysis choice and oftentimes might be confounded by a change of the PSD slope. The manuscript is well written and it will be of interest to a diverse group of neuroscientists. A few minor aspects should be addressed, but the manuscript already constitutes an excellent resource for future investigations.

Reviewer 2:

The authors use a combination of simulation and EEG data analysis to demonstrate that band ratio measures cannot be interpreted as changes in oscillatory power at different frequency bands. Rather, they show that the estimation of band ratios can be affected by oscillatory power as well as the aperiodic signal of the power spectrum. In order to avoid mixing periodic and aperiodic signals as band ratio do, the authors propose to parametrize the power spectra into periodic and aperiodic components and relate them to features of interest. This is an excellent paper that may have a significant impact for the understanding of the relationship between oscillations, aperiodic signals and cognition. I have just a few questions about the analysis and the discussion:

1. The authors argue that aperiodic activity is a confound in the computation of band ratios using total-band power: as a consequence, changes in band ratios may reflect changes in periodic, aperiodic signal or both. Furthermore, the aperiodic signal is know to vary, not only between individuals, but also within individuals, i.e. on a moment-to-moment basis. It would be interesting to see how total-band ratio is related to the aperiodic signal on a trial-by-trial basis. You could for example bin the trials (i.e. segments of the EEG resting state) based on total-band ratio and calculate the periodic and aperiodic features for the different bins, or vice-versa. I believe this analysis would strengthen the already compelling evidence that total-band ratio mixes periodic and aperiodic signals.

2. I wonder if the authors thought about computing a cleaner estimate of band ratio using the flattened power spectrum (i.e. after removal of 1/f aperiodic signal). If the periodic estimate of band ratio has specific functional properties, then it may be related to cognitive state, disease, age, etc.

3. I wonder if you could elaborate a little bit more about the reasoning behind the analysis of band ratio rather than the power in individual frequency bands in previous literature, discussed on page 22 (lines: 326-334). Has any of the studies you mentioned systematically compared the effects of band ratio versus oscillatory power in specific frequencies? For example, if theta / alpha and alpha / beta ratios likely reflect alpha power, as suggested in your manuscript, then I wouldn't expect a benefit of analyzing band ratio, over simply analyzing alpha-band power.

4. In addition to band ratio, another measure that compares the power in different bands is the correlation between power at different frequencies. Specifically, many studies have reported a correlation between alpha and gamma power: the stronger the alpha, the weaker the gamma power. I wonder if the authors could briefly discuss the interpretation of these power correlations. Is the alpha/gamma power correlation affected by the aperiodic signal in the same way that band ratio is? Can the author suggest an analysis to properly compare power at different frequencies?

5. on page 12 (line 186-9), please include how many channels were rejected and interpolated per subject (average {plus minus}SEM) and how many channels were analyzed.
